# A Pilot Study on the Association of Lead, 8-Hydroxyguanine, and Malondialdehyde Levels in Opium Addicts’ Blood Serum with Illicit Drug Use and Non-Addict Persons

**DOI:** 10.3390/ijerph19159110

**Published:** 2022-07-26

**Authors:** Farzaneh Allahdinian Hesaruiyeh, Saeed Rajabi, Mohadeseh Motamed-Jahromi, Mohammad Sarhadi, Michelle L. Bell, Razieh Khaksefidi, Somayeh Sarhadi, Leili Mohammadi, Kamal Dua, Amin Mohammadpour, Paolo Martelletti

**Affiliations:** 1 Department of Toxicology, Faculty of Pharmacy, Shahreza Branch, Islamic Azad University, Shahreza P.O. Box 311-86145, Iran; f.allahdinian@zaums.ac.ir; 2 Clinical Core Laboratory, Ali ibn Abi Talib Hospital Complex, Zahedan University of Medical Sciences, Zahedan 98167-43463, Iran; 3 Student Research Committee, School of Health, Shiraz University of Medical Sciences, Shiraz 71348-45794, Iran; saeedrajabi@sums.ac.ir (S.R.); r_khaksefidi@sums.ac.ir (R.K.); 4 Nursing School, Fasa University of Medical Science, Fasa 74616-86688, Iran; motamed@fums.ac.ir; 5 Cellular and Molecular Research Center, Research Institute of Cellular and Molecular Sciences in Infectious Diseases, Zahedan University of Medical Sciences, Zahedan 98167-43463, Iran; sarhadi@zaums.ac.ir; 6 School of Forestry and Environmental Studies, Yale University, New Haven, CT 06520, USA; michelle.bell@yale.edu; 7 Department of Biology, Faculty of Sciences, Hamedan Branch, Islamic Azad University, Hamedan 15847-43311, Iran; s.sarhadi@iauh.ac.ir; 8 Environmental Health, Infectious Diseases and Tropical Medicine Research Center, Research Institute of Cellular and Molecular Sciences in Infectious Diseases, Zahedan University of Medical Sciences, Zahedan 98167-43463, Iran; leili.mohamadi@zaums.ac.ir; 9 Centre for Inflammation, Centenary Institute, Sydney, NSW 2050, Australia; kamal.dua@uts.edu.au; 10 Priority Research Centre for Healthy Lungs, Hunter Medical Research Institute (HMRI) & School of Biomedical Sciences and Pharmacy, University of Newcastle, Callaghan, NSW 2308, Australia; 11 Discipline of Pharmacy, Graduate School of Health, University of Technology Sydney, Ultimo, NSW 2007, Australia; 12 Department of Clinical and Molecular Medicine, Sapienza University of Rome, 00185 Rome, Italy

**Keywords:** addicts, lead, 8-hydroxyguanine, malondialdehyde, blood serum

## Abstract

While a large body of literature has shown the health problems of illicit drug use, research is needed on how substance abuse impacts DNA damage and contaminants in blood, especially given Pb-contaminated opium. This pilot study aimed to evaluate the levels of lead (Pb), 8-hydroxy di-guanine (8-oxo-Gua), and malondialdehyde (MDA) in the blood serum of opium addicts and non-addict people. The current study is a case–control study with a cross-sectional design. A sample of 50 opium-addicted and non-addict adults were chosen for this study using convenience and random sampling methods. Participants were divided into two groups: addicts and non-addicts. The atomic absorption spectroscopy method was used to measure the quantity of Pb, and the Enzyme-Linked Immunosorbent Assay (ELISA) method was used to measure the amount of 8-oxo-Gua and MDA. The data were analyzed using an independent t-test. The results show that the amount of Pb in the blood serum of addicted women and men was higher than levels in non-addict men and women, for the study participants (*p*-value = 0.001). Blood levels were not significantly different between addicts and non-addicts for men or women for 8-oxo-Gua (*p*-value = 0.647 for women and *p*-value = 0.785 for men) and MDA (*p*-value = 0.867 for women and *p*-value = 0.995 for men). In general, addicts’ blood Pb levels were found to be substantially higher than those of normal non-addict persons in this pilot study. As a result, testing for blood Pb levels in addicts may be informative in instances when symptoms are inconclusive.

## 1. Introduction

Drug addiction is one of the most visible psychological and social diseases that may quickly disrupt the individual, familial, social, and cultural life of a society, and rising addiction to stimulants and pharmaceutical drugs can modify mood and behavior. Addiction can adversely affect the workforce, jeopardize individual motivation and interests, and deplete the monetary and spiritual resources of individuals and communities. Substance misuse and addiction are multifaceted illnesses having biological, psychological, social, and spiritual origins and consequences [1]. The rise in illicit drug use has become a major concern for all communities. Substance addiction and misuse have evolved into a socio-therapeutic concern [2]. Trends in usage include the decrease in the mean age of consumers [3]. The frequency of occupational lead (Pb) poisoning in adults has declined in recent years due to increased workplace safety and improving housing characteristics [4], although Pb-contaminated drugs could be an emerging source.

A comprehensive investigation of the phenomena of addiction reveals that illicit drug usage has existed in past societies and that public knowledge is continually growing as a result of scientific discoveries and education campaigns [5]. In addition, the number of illicit drugs available on the market has expanded. As access to abused substances and the types of substances evolve, many, particularly youth and those in middle age, are becoming increasingly active in illicit drug use. The number of illicit drug users has been increasing, posing a substantial public health burden [6].

Due to the consumption of Pb-contaminated opium, a new kind of Pb poisoning is progressively arising. In opium users, pathological signs such as abdominal pain, neuropathy, and anemia have been noted. These findings might imply Pb toxicity in these people, emphasizing the significance of screening them for Pb poisoning [7]. Lead is a heavy metal that can cause acute or chronic poisoning in humans [8]. Industrial pollutants, soils, automobile emissions, and contaminated foods are the most common causes of Pb exposure. Poisoning can result from contact with Pb from any of these routes, whether it is through the mouth, inhalation, or the skin [9,10].

One of the most prevalent DNA lesions generated by reactive oxygen species is 8-hydroxy-di-guanine (8-oxo-Gua). This can result in adenine-incompatible pairings on the genome, such as G to T and C to A [11]. The 8-oxo-Gua enzyme is produced by damaged sequences of DNA as a result of drug usage, and it can be utilized as a biomarker to monitor drug use [12].

Malondialdehyde (MDA) is an active and highly reactive aldehyde molecule formed in the human body by the peroxidation of unsaturated fatty acids [13]. As a result, the degree of fat peroxidation may be recognized and utilized as a marker to quantify the level of oxidative stress in an organism by measuring the quantity of MDA in different biological samples [14]. MDA is an active and highly reactive chemical that assaults the function of other molecules while connecting with them covalently and forcefully, altering molecular function and, eventually, cell function [13,14]. The pathophysiology of abused substances is associated with oxidative stress [15]. The oxidative stress process causes lipid and nucleic acid oxidation. As a significant endogenous result of lipid peroxidation, MDA is an essential biomarker of oxidative stress and has been related to illicit drug use [15].

Human biomonitoring is concerned with the detection and distribution of contaminants and related metabolites in the human body, as well as associated disorders. To measure exposure to chemicals or related metabolites, biomonitoring in biological matrices such as urine, serum, and blood can be used [16]. Pollutant concentrations in serum may be indicative of their circulation across organs in the human body. As a result, a high-throughput extraction approach based on a tiny volume of serum sample is required, as well as an instrumental analysis method capable of detecting minuscule levels of contaminants with high precision [17,18,19].

Scientific evidence is needed on whether levels of Pb, 8-oxo-Gua, and MDA are elevated by substance abuse. Due to the limited number of samples, this study was conducted as a pilot study. In this pilot study, the levels of Pb, 8-oxo-Gua, and MDA were compared between a patient group of opium addicts referred to the Zahedan addiction and detoxification clinic, and a non-addict control group, to determine the possible role of Pb-infected opium abuse. Due to the great prevalence of opium addiction and the associated individual and social complications, this study was conducted to evaluate whether substance abuse is introducing new sources of Pb exposure and acute or chronic poisoning.

## 2. Materials and Methods

### 2.1. Study Population

The current pilot study is a case–control analysis with a cross-sectional strategy that was conducted in 2020 [13]. Addicts and non-addict persons residing in Zahedan, Iran, constituted the study population. In the center of the addiction and detoxification clinic (Life Revitalization Center) in Zahedan, Iran, a sample was randomly selected from the patient population and divided into two groups, an experimental group (25 participants with addiction) and a control group (25 participants without addiction), using the accessible method. In the accessible method, the available people in the community are randomly selected [20]. The control group comprised 25 non-addict people who were as similar to the experimental group (addicts) as feasible in terms of age and socioeconomic status (education level and employment), but were not addicts.

Inclusion criteria for the addict group included a history of opium use for at least 3 years, except for users of tramadol, acetaminophen codeine, and diphenoxylate, and those aged from 20 to 50 years old. Opium use was defined as the self-reported use of opium. The addicts in this pilot study are persons who use drugs often and every day, and who suffer from physical pains, lethargy, and hangovers if they do not use them. Exclusion criteria included a history of Pb poisoning; a history of occupational exposure to Pb such as battery making, soldering, dyeing, pottery, and plumbing; and specific physical and psychological problems such as physical disability, paralysis, autism, depression, and mental retardation. Importantly, “addicts” in this pilot study refers to opium addicts specifically. To generate a control group, all addict participants were requested to recommend a member of their first-degree family or, if that was not feasible, a member of their second-degree family who was the same sex as the participant and with an age gap of fewer than 5 years. Then, using a written questionnaire, all demographic information, health status, and lifestyle information from both groups were collected.

### 2.2. Sample Preparation and Analysis

To measure blood Pb, 8-oxo-Gua, and MDA levels, 5 mL of venous blood from the study groups was collected in sterile tubes for blood (containing EDTA as an anticoagulant), and atomic absorption spectroscopy (AAS), Enzyme-Linked Immunosorbent Assay (ELISA) Kit 8-hydroxy-di-guanine, and ELISA Kit malondialdehyde was used to investigate the amounts of Pb, 8-oxo-Gua, and MDA in the blood serum of these participants.

The amount of radiation absorbed by the atoms of the element provides the basis for atomic-absorption spectroscopy, and the amount of radiation absorbed is proportional to the element’s concentration [21]. A monochromatic beam is first generated in this procedure using hollow-cathode lamps or electric discharges. To accomplish this, the sample must first be dissolved in a specific solvent before being injected into the flame via a sprayer, where it becomes a free atom. These atoms pass some of the monochromatic beams after they have passed. They are quickly absorbed, and the intensity of the radiation is reduced [21].

The ELISA kit, known as Enzyme Immunoassay (EIA), is a biochemical technique that can be used to check for the existence of an antigen or antibody in a sample. It is one of the most potent laboratory procedures for diagnosing immune system problems. The amount of antigen fixed on the surface of a container or plate is then coupled to the antigen by a specific antibody that is dropped on the surface in a simple ELISA method. Antigens and antibodies are routinely detected using ELISA. Thus, one of these two compounds is fixed in a solid bed and utilized to detect another, but any pair of substances that tend to have high binding strength, such as an antigen and antibody, can also be used [22].

### 2.3. Quality Assurance and Quality Control (QA/QC)

**Figure 1:**
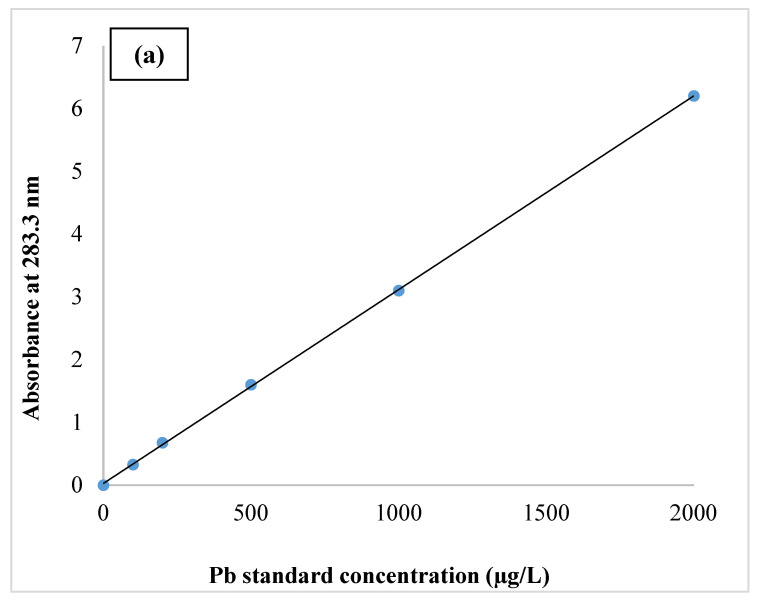

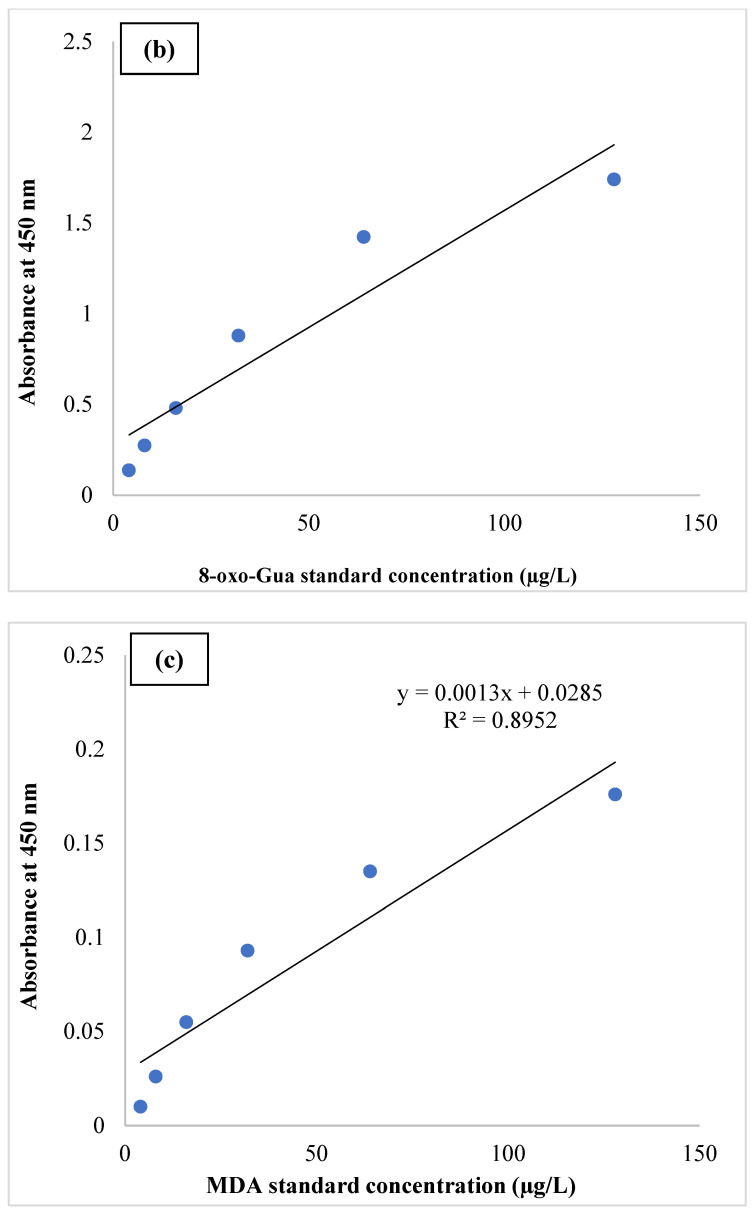
Standard curves of Pb (**a**), 8-oxo-Gua (**b**), and MDA (**c**).

The standard Pb curve (Figure 1a) was plotted using standard Pb solutions of 0, 100, 200, 500, 1000, and 2000, (µg/L) ppb, and the absorption for each concentration was at 283.3 nm. The 2000 ppb standard was prepared first, followed by the rest of the standard solutions. Then, 0.20 mL of the manufactured standard 1000 µg/mL Pb was transferred under the hood into a 100 mL balloon, then made up to volume with 1% *v*/*v* nitric acid and 65% purity, and mixed well with a mechanical stirrer to prepare the 2000 ppb standard [23]. Similarly, standard curves for 8-oxo-Gua (Figure 1b) and MDA (Figure 1c) with standard concentrations of 0, 10, 20, 30, 60, and 130 ppb were also plotted at 450 nm. Pb, 8-oxo-Gua, and MDA all have R2 values between 0.890 and 0.999, indicating a high correlation.

### 2.4. Statistical Analysis

The Kolmogorov–Smirnov analysis was applied with SPSS-v.20 (SPSS Inc., Chicago, IL, USA) statistical software to determine that the collected data of the individuals had a normal distribution, and an independent t-test was utilized to compare the results of the opium addict and non-addict persons.

## 3. Results and Discussion

Table 1 shows that the majority of addicts (60%) and non-addicts (52%) were males. Additionally, most addicts (76%) and non-addicts (72%) are between 20 and 40 years old. Moreover, over half of both addicted and non-addicted individuals had an academic qualification (e.g., Associate’s degree, BSc., MSc., and Ph.D.), and most addicts and non-addicts were employed, according to the study’s findings. A total of 16% of non-addicts had a history of drug use, compared to 36% of addicts with a history of 3 years, 28% with a history of 4 years, 20% with a history of 5 years, and 16% with a history of more than 5 years. The majority of addicts in the survey were opium users, with 20% of non-addicts having used illicit drugs including opium, heroin, marijuana, and other substances.

Table 2 shows that the frequency of Pb in the blood serum of addicts is higher than the level of Pb in the blood serum of non-addict participants by concentrations of more than 25 mg/dL. When comparing the amounts of 8-oxo-Gua and MDA in addicts and non-addict participants, there is no substantial difference.

As shown in Table 3, the average Pb concentration in addicted and non-addict women and men significantly differs. The p-value was smaller than 0.05, indicating statistical significance. In other words, the concentration of Pb in addicted women’s blood serum is higher than that in non-addict women. The mean concentration of 8-oxo-Gua in addicted and non-addict women and men, on the other hand, did not differ significantly (*p*-value = 0.647 for women, 0.785 for men). Furthermore, there was no significant difference in the MDA concentrations of addicted and non-addict women and men (*p*-value = 0.867 for women, *p*-value = 0.995 for men). These findings indicate that illicit drug use is strongly and positively associated with increases in the concentration of Pb in blood serum, but not with a rise in the concentrations of 8-oxo-Gua and MDA.

Occupational Pb poisoning has declined as the primary source of Pb toxicity in recent years, but new non-occupational origins have developed [24,25]. In the past few decades, the negative consequences of opioids have become a prominent problem in the study region of Iran. Nevertheless, multiple case reports have been published about opium-related Pb toxicity [26,27]. Lead is poisonous to various body systems and can manifest itself in several different ways. Colicky abdominal symptoms, bowel problems, decreased appetite, vomiting, as well as other organ-related symptoms such as joint pain, muscle aches, dizziness, sexual dysfunction, sleeplessness, nervousness, tiredness, anemia, renal disease, confusion, encephalitis, and convulsions are all major signs of Pb poisoning [28,29,30,31]. Abdominal discomfort and anemia are common symptoms of opium-related Pb poisoning. Constipation is common among opium users. Intense abdominal pain has a widely varying diagnosis, and Pb toxicity should be evaluated as an additional differential diagnosis in addicts who report stomach pain in addition to the main potential illness [32,33]. Similarly, Farzaneh et al. found similar outcomes in their research. According to the findings of this study, the concentration of blood Pb in addicts is higher than in non-addict persons [34].

Reactive oxygen species (ROS) and other oxidizing species attack biomolecules directly, causing damage to lipids, proteins, and, if contained within the cell nucleus, DNA. DNA damage can become severe in the presence of frequent and prolonged intra-nuclear ROS, and excessive DNA damage leads to genomic instability, which can lead to carcinogenesis [35,36]. Endogenous ROS, such as the hydroxyl radical (HO•) produced during normal oxidative metabolism, can cause chemical changes in purines and pyrimidines, affecting gene stability [37,38]. Due to its great reactivity, HO• formed in a distant cell compartment is unlikely to spread into the cell nucleus, and it has been postulated that H_2_O_2_ operates as a diffusible latent type of HO• that combines with a metal ion in the proximity of a DNA molecule to produce the oxidant component. Others have proposed that lipid peroxidation molecules serve as a bridge between endogenous metabolic products, xenobiotic agent-induced changes, and DNA impacts. In a cell with replicative capacity, every oxidative damage that is not treated might become a permanent mutation, increasing the risk of carcinogenesis [39,40,41,42]. The particulate matter in cigarette smoke includes stable ROS with extended half-lives, and certain species may be found in the gas phase of cigarette smoke. These reactive species may cause harm by interacting directly with tissues and cell membranes. Tissue injury involves inflammation, which leads to the release of more oxidative species, resulting in a redox state of disequilibrium. Because of the mispairing of 8-oxo-Gua with adenine, the interaction between ROS and DNA might result in the production of oxidized DNA bases such as 8-oxo-Gua, which can trigger base alterations [37,43,44]. Moreover, certain mechanisms involved in DNA damage and the anti-oxidative defense system were shown to have no effect from smoking. 8-oxo-Gua concentrations in peripheral blood lymphocytes have similarly generated inconsistent findings, with some studies indicating higher amounts in smokers than non-smokers [45,46] and others showing no increase [47,48].

The activation of oxidative stress in biological processes can result in lipid peroxidation, which can change cell structure and lipid metabolism in the human biological system. A rise in oxidative stress may have negative repercussions. In many illnesses, tumors, and malignancies, the oxidative alteration of DNA, protein, and lipids plays a critical role [49,50]. Cell damage and death are caused by oxidative stress, which leads to pathologies such as cardiovascular disease, inflammatory diseases, neuropathies, acquired immune deficiency syndrome (AIDS), renal disease, diabetes mellitus, cancer [51], Parkinson’s disease [52], atherosclerosis, Alzheimer’s, rheumatoid arthritis, schizophrenia, thalassemia, male and female infertility, hypertension, muscle degeneration, asthma, heart failure, and many other disorders [53]. Drug-induced oxidative stress adds to cytotoxicity. The antioxidant defense system is weakened by persistent drug misuse; in illicit drug addicts, a rise in oxidative stress and a lack of antioxidant defense mechanisms eventually disturb the physiological balance and lead to diseases [15]. MDA is a crucial indicator for determining lipid peroxidation levels. Our findings were similar to the results of Najafi’s study indicating little difference in serum MDA levels between illicit drug users and non-users [54].

## 4. Study Limitations and Strengths

One of the study’s strengths is the evaluation of Pb, 8-oxo-Gua, and MDA in opium addicts as a special group. However, no previous research has been conducted on the association between Pb, 8-oxo-Gua, and MDA and illicit drug usage in Iranian addicts. A major limitation of this research is that it is cross-sectional in design and does not have a temporal element. Furthermore, given the pilot study design, the analysis cannot account for illicit drug variance. The results are based on opium addicts from addiction and detoxification treatment in Zahedan, Iran, and may not be generalizable to those with addictions to other substances, those who are less likely to seek treatment, and those in other communities. Other limitations include the control group, which may include addicts, and the sample size. Information from the questionnaire was self-reported. For these reasons, while results are informative, they should be treated with caution and should not be used to confidently identify causal associations.

## 5. Conclusions

In this pilot study, the amount of Pb in blood serum was higher in the opium addict groups, for both men and women, than in the control group, and the difference was statistically significant. Furthermore, the distribution of participant characteristics (e.g., age) was roughly similar between the opium addicts and the control group, although the addicts overall had lower education levels and more unemployment, with the main distinction being the usage of illicit drugs. As a result, the higher blood Pb levels in the addicted men and women might be the result of Pb drug contamination. This is because the main difference between the two groups is the addicts’ history of opium use, as well as the recent discovery of Pb-contaminated opium as one of the rising sources of exposure to this toxin in the study region, due to the likely misuse of Pb-contaminated opium. The findings of this pilot study also corroborate numerous reports pointing to Pb poisoning as a cause of nonspecific symptoms in opium addicts, such as colic abdominal pain, nausea, vomiting, weight loss, anemia, neuropathy, and so on, indicating the need to screen blood Pb levels in opium addicts to avoid more serious complications. The restricted results of this pilot study necessitate further investigation by other researchers, and this pilot study can serve as the foundation for future pertinent research.

## Figures and Tables

**Table 1:** The distribution of parameters frequency.

Variable	Frequencies (%)		Std. Deviation	
	Addict	Non-Addict	Addict	Non-Addict
Sex				
Male	15 (60%)	13 (52%)	0.50	0.51
Female	10 (40%)	12 (48%)		
Age groups (years old)				
20–30	7 (28%)	9 (36%)	0.73	0.81
30–40	12 (48%)	9 (36%)		
40–50	6 (24%)	7 (28%)		
Education				
Illiterate	1 (4%)	7 (28%)	0.59	0.90
Non-academic (high school diploma)	11 (44%)	3 (4%)		
Academic (e.g., Associate’s degree, BSc., MSc., and Ph.D.)	13 (52%)	15 (60%)		
Job				
Unemployed	3 (12%)	8 (32%)	0.45	0.69
Employee	20 (80%)	13 (52%)		
Retired	2 (8%)	4 (16%)		
History of illicit drug use				
Never	0 (0%)	20 (80%)	1.11	0.52
3 years	9 (36%)	4 (16%)		
4 years	7 (28%)	1 (4%)		
5 years	5 (20%)	0 (0%)		
>5 years	4 (16%)	0 (0%)		
Drug type				
Opium	11 (44%)	1 (4%)	1.64	1.57
Heroin	4 (16%)	1 (4%)		
Cocaine	0 (0%)	0 (0%)		
Marijuana	6 (24%)	1 (4%)		
Other	4 (16%)	2 (8%)		

**Table 2:** The frequency of blood Pb, 8-oxo-Gua, and MDA levels in addict and non-addict participants.

Variable	Frequency (%)		Std. Deviation	
	Addict	Non-Addict	Addict	Non-Addict
Pb (µg/dL)				
<25	14 (28%)	9 (18%)	0.94516	1.09240
25–35	7 (14%)	8 (16%)		
35–45	2 (4%)	4 (8%)		
>45	2 (4%)	4 (8%)		
8-oxo-Gua (ng/mL)			1.43991	1.38082
64	10 (20%)	9 (18%)		
32	5 (10%)	6 (12%)		
16	4 (8%)	5 (10%)		
8	3 (6%)	2 (4%)		
4	3 (6%)	3 (6%)		
MDA (ng/mL)				
64	8 (16%)	9 (18%)		
32	7 (14%)	7 (14%)	1.25433	1.26754
16	4 (8%)	5 (10%)		
8	5 (10%)	2 (4%)		
4	1 (2%)	2 (4%)		

**Table 3:** Mean concentration of Pb, 8-oxo-Gua, and MDA in blood serum of addict and non-addict participants.

Variable Indicators		Mean Concentration (µg/dL)		Std. Deviation		*p*-Value * df (f)
		Addict	Non-Addict	Addict	Non-Addict	
Pb	Male	22.78	10.11	13.79	4.19	0.001 48 (4.87)
	Female	21.76	8.84	12.36	3.29	0.001 48 (4.66)
8-oxo-Gua	Male	22.57	22.90	11.55	11.46	0.785 48 (0.88)
	Female	23.11	23.76	14.76	14.66	0.647 48 (0.94)
MDA	Male	25.64	25.55	12.77	12.75	0.995 48 (0.97)
	Female	24.84	23.98	13.34	13.67	0.867 48 (0.57)

* *p*-value is significant at the 0.05 level.

## Data Availability

The original contributions presented in the study are included in the article; further inquiries can be directed to the corresponding author.

## References

[1] Heilig M., MacKillop J., Martinez D., Rehm J., Leggio L., Vanderschuren L.J. (2021). Addiction as a brain disease revised: Why it still matters, and the need for consilience. Neuropsychopharmacology.

[2] Koob G.F. (2021). Drug addiction: Hyperkatifeia/negative reinforcement as a framework for medications development. Pharmacol. Rev..

[3] Lee M.Y., Lee B.H., Kim H.Y., Yang C.H. (2021). Bidirectional role of acupuncture in the treatment of drug addiction. Neurosci. Biobehav. Rev..

[4] Takahashi T.T., Ornello R., Quatrosi G., Torrente A., Albanese M., Vigneri S., Guglielmetti M., Maria De Marco C., Dutordoir C., Colangeli E. (2021). Medication overuse and drug addiction: A narrative review from addiction perspective. J. Headache Pain.

[5] Loganathan K., Ho E.T.W. (2021). Value, drug addiction and the brain. Addict. Behav..

[6] Calpe-López C., Martínez-Caballero M.A., García-Pardo M.P., Aguilar M.A. (2022). Resilience to the effects of social stress on vulnerability to developing drug addiction. World J. Psychiatry.

[7] Antonini G., Palmieri G., Millefiorini E., Spagnoli L., Millefiorini M. (1989). Lead poisoning during heroin addiction. Ital. J. Neurol. Sci..

[8] Soltaninejad K., Shadnia S. (2018). Lead poisoning in opium abuser in Iran: A systematic review. Int. J. Prev. Med..

[9] Soltaninejad K., Flückiger A., Shadnia S. (2011). Opium addiction and lead poisoning. J. Subst. Use.

[10] Nasab H., Rajabi S., Eghbalian M., Malakootian M., Hashemi M., Mahmoudi-Moghaddam H. (2022). Association of As, Pb, Cr, and Zn urinary heavy metals levels with predictive indicators of cardiovascular disease and obesity in children and adolescents. Chemosphere.

[11] Kroese L.J., Scheffer P.G. (2014). 8-hydroxy-2′-deoxyguanosine and cardiovascular disease: A systematic review. Curr. Atheroscler. Rep..

[12] Schmidt A.J., Clement H.W., Gebhardt S., Hemmeter U.M., Schulz E., Krieg J.C., Kircher T., Heiser P. (2010). Impact of psychostimulants and atomoxetine on the expression of 8-hydroxyguanine glycosylase 1 in human cells. J. Neural Transm..

[13] Rybus-Kalinowska B., Zwirska-Korczala K., Kalinowski M., Kukla M., Birkner E., Jochem J. (2008). Activity of antioxidative enzymes and concentration of malondialdehyde as oxidative status markers in women with newly diagnosed Graves-Basedow disease and after thiamazole therapy leading to euthyroidism. Polskie Archiwum Medycyny Wewnetrznej.

[14] Rybus-Kalinowska B., Żwirska-Korczala K., Kalinowski M., Kukla M., Birkner E., Jochem J. (2009). Activity of antioxidative enzymes and concentration of malondialdehyde as oxidative status markers in women with non-autoimmunological subclinical hyperthyroidism. Endokrynologia Polska.

[15] Luan X., Chen H., Qiu H., Shen H., Zhao K., Ren W., Gu Y., Su H., Zhang J., Lv D. (2018). Association between serum malondialdehyde levels and depression during early methamphetamine withdrawal. Neurosci. Lett..

[16] Polachova A., Gramblicka T., Bechynska K., Parizek O., Parizkova D., Dvorakova D., Honkova K., Rossnerova A., Rossner P., Sram R.J. (2021). Biomonitoring of 89 POPs in blood serum samples of Czech city policemen. Environ. Pollut..

[17] Nasab H., Mirzaee M., Hashemi M., Rajabi S. (2022). Measurement of Urinary Triclocarban and 2, 4-Dichlorophenol Concentration and Their Relationship with Obesity and Predictors of Cardiovascular Diseases among Children and Adolescents in Kerman, Iran. J. Environ. Public Health.

[18] Nasab H., Rajabi S., Mirzaee M., Hashemi M. (2022). Association of urinary triclosan, methyl triclosan, triclocarban, and 2,4-dichlorophenol levels with anthropometric and demographic parameters in children and adolescents in 2020 (case study: Kerman, Iran). Environ. Sci. Pollut. Res..

[19] Nasab H., Mirzaee M., Ebrahimpour K., Hashemi M. (2021). Association of urinary triclosan and methyl-triclosan levels with predictive indicators of cardiovascular disease and obesity in children and adolescents in 2020 (case study: Kerman, Iran). Environ. Health Eng. Manag. J..

[20] Asiamah N., Mensah H.K., Oteng-Abayie E.F. (2017). General, target, and accessible population: Demystifying the concepts for effective sampling. Qual. Rep..

[21] Suljević D., Handžić N., Fočak M., Lasić I., Sipović F., Sulejmanović J., Begić S., Alijagic A. (2021). Lead exposure influences serum biomarkers, hepatocyte survival, bone marrow hematopoiesis, and the reproductive cycle in Japanese quails. Biol. Trace Elem. Res..

[22] Malavika L., Mitra P., Goyal T., Sharma S., Purohit P., Sharma P. (2021). Association of blood lead level with neurobehavior and neurotransmitter expressions in Indian children. Toxicol. Rep..

[23] Park E., Kim J., Kim B., Park E.Y. (2021). Association between environmental exposure to cadmium and risk of suspected non-alcoholic fatty liver disease. Chemosphere.

[24] Masoudi M., Zali M.R., Ehsani M., Mohammadalizadeh A.H., Aiassofi K., Aghazadeh R., Shavakhi A., Soumi M., Antikchi M.H., Yazdani S. (2006). Abdominal pain due to lead-contaminated opium: A new source of inorganic lead poisoning in Iran. Arch. Iran. Med..

[25] Busse F.P., Fiedler G.M., Leichtle A., Hentschel H., Stumvoll M. (2008). Lead poisoning due to adulterated marijuana in Leipzig. Deutsches Ärzteblatt Int..

[26] Vossoughinia H., Pourakbar A., Esfandiari S., Sharifianrazavi M. (2016). Severe abdominal pain caused by lead toxicity without response to oral chelators: A case report. Middle East, J. Dig. Dis..

[27] Beigmohammadi M.T., Aghdashi M., Najafi A., Mojtahedzadeh M., Karvandian K. (2008). Quadriplegia due to lead-contaminated opium. MEJ ANESTH.

[28] Tabrizi R., Sarihi S., Moazzen F., Hosseini-Bensenjan M., Malekpour F., Asadikaram G., Momeni-Moghaddam M.A., Akbari H. (2021). A systematic review and meta-analysis on blood lead level in opium addicts: An emerging health threat. Biol. Trace Elem. Res..

[29] Khatibi-Moghadam H., Khadem-Rezaiyan M., Afshari R. (2016). Comparison of serum and urine lead levels in opium addicts with healthy control group. Hum. Exp. Toxicol..

[30] Ahmadinejad M., Ahmadipour M., Divsalar K. (2019). Blood lead level in opiate addicts hospitalized in the intensive care unit of a trauma referral center in Kerman, Iran. Addict. Health.

[31] Shafikhani A.A., Kazemifar A.M. (2019). Comparison of blood lead levels between oral and inhalation opium addicts and its relationship with hematological parameters. Indian J. Forensic Med. Toxicol..

[32] Shojaeepour S., Fazeli M., Mandegary A., Sayed-Mirzaei S.M., Ahmadi N., Saeedi A., Oghabian Z. (2017). Evaluation of oxidative stress in combination therapy with d-penicillamine and n-acetylcysteine (NAC) in lead poisoning in opium addicts. Asia Pac. J. Med. Toxicol..

[33] Shahramian I., Noori N.M., Afshari M., Delaramnasab M., Bazi A., Abdollahi M. (2019). The clinical relevance of elevated blood lead levels in opium addicts with severe abdominal pain. Arch. Psychiatry Res. Int. J. Psychiatry Relat. Sci..

[34] Farzaneh E., Habibzadeh A., Mehrpour O. (2017). Lead toxicity among oral opium addicts with abdominal pain: A case series of 17 cases. Prof. RK Sharma.

[35] Charames G.S., Bapat B. (2003). Genomic instability and cancer. Curr. Mol. Med..

[36] Lowe F.J., Luettich K., Gregg E.O. (2013). Lung cancer biomarkers for the assessment of modified risk tobacco products: An oxidative stress perspective. Biomarkers.

[37] Valavanidis A., Vlachogianni T., Fiotakis K. (2009). Tobacco smoke: Involvement of reactive oxygen species and stable free radicals in mechanisms of oxidative damage, carcinogenesis and synergistic effects with other respirable particles. Int. J. Environ. Res. Public Health.

[38] Valko M., Izakovic M., Mazur M., Rhodes C.J., Telser J. (2004). Role of oxygen radicals in DNA damage and cancer incidence. Mol. Cell. Biochem..

[39] Voulgaridou G.-P., Anestopoulos I., Franco R., Panayiotidis M.I., Pappa A. (2011). DNA damage induced by endogenous aldehydes: Current state of knowledge. Mutat. Res. Fundam. Mol. Mech. Mutagenesis.

[40] Marnett L.J. (2002). Oxy radicals, lipid peroxidation and DNA damage. Toxicology.

[41] Tudek B., Swoboda M., Kowalczyk P., Oliński R. (2006). Modulation of oxidative DNA damage repair by the diet, inflammation and neoplastic transformation. J. Physiol. Pharmacol. Off. J. Pol. Physiol. Soc..

[42] Knaapen A.M., Güngör N., Schins R.P., Borm P.J., Van Schooten F.J. (2006). Neutrophils and respiratory tract DNA damage and mutagenesis: A review. Mutagenesis.

[43] Faux S.P., Tai T., Thorne D., Xu Y., Breheny D., Gaca M. (2009). The role of oxidative stress in the biological responses of lung epithelial cells to cigarette smoke. Biomarkers.

[44] Smith C.J., Perfetti T.A., King J.A. (2006). Perspectives on pulmonary inflammation and lung cancer risk in cigarette smokers. Inhal. Toxicol..

[45] Lodovici M., Caldini S., Luceri C., Bambi F., Boddi V., Dolara P. (2005). Active and passive smoking and lifestyle determinants of 8-oxo-7, 8-dihydro-2′-deoxyguanosine levels in human leukocyte DNA. Cancer Epidemiol. Prev. Biomark..

[46] Yao Q.H., Mei S.R., Weng Q.F., Zhang P.D., Yang Q., Wu C.Y., Xu G.W. (2004). Determination of urinary oxidative DNA damage marker 8-hydroxy-2′-deoxyguanosine and the association with cigarette smoking. Talanta.

[47] Nia A.B., Van Schooten F.J., Schilderman P.A.E.L., De Kok T.M.C.M., Haenen G.R., Van Herwijnen M.H.M., Van Agen E., Pachen D.M.F.A., Kleinjans J.C.S. (2001). A multi-biomarker approach to study the effects of smoking on oxidative DNA damage and repair and antioxidative defense mechanisms. Carcinogenesis.

[48] van Zeeland A.A., de Groot A.J., Hall J., Donato F. (1999). 8-Hydroxydeoxyguanosine in DNA from leukocytes of healthy adults: Relationship with cigarette smoking, environmental tobacco smoke, alcohol and coffee consumption. Mutat. Res./Genet. Toxicol. Environ. Mutagenesis.

[49] Shadnia S., Gorgzadeh N., Soltaninejad K., Abdollahi M., Motevalian S. (2017). Status of Total Antioxidant Capacity and Malondialdehyde Level in Methamphetamine Addicts: A Cross Sectional Study. Int. J. Med. Toxicol. Forensic Med..

[50] Karajibani M., Montazerifar F., Feizabad A.K. (2017). Study of oxidants and antioxidants in addicts. Int. J. High Risk Behav. Addict..

[51] Macotpet A., Suksawat F., Sukon P., Pimpakdee K., Pattarapanwichien E., Tangrassameeprasert R., Boonsiri P. (2013). Oxidative stress in cancer-bearing dogs assessed by measuring serum malondialdehyde. BMC Vet. Res..

[52] Karatas F., Karatepe M., Baysar A. (2002). Determination of free malondialdehyde in human serum by high-performance liquid chromatography. Anal. Biochem..

[53] Rahman T., Hosen I., Islam M.T., Shekhar H.U. (2012). Oxidative stress and human health. Adv. Biosci. Biotechnol..

[54] Najafi K., Ahmadi S., Rahpeyma M., Khazaie H., Vaisi-Raygani A., Moini A., Kiani A. (2017). Study of serum malondialdehyde level in opioid and methamphetamine dependent patients. Acta Med. Iran..

